# BI2536 induces mitotic catastrophe and radiosensitization in human oral cancer cells

**DOI:** 10.18632/oncotarget.25035

**Published:** 2018-04-20

**Authors:** Chieh-Yuan Cheng, Chung-Ji Liu, Yu-Chuen Huang, Shu-Hua Wu, Hsu-Wei Fang, Yu-Jen Chen

**Affiliations:** ^1^ Graduate Institute of Engineering Technology, National Taipei University of Technology, Taipei 10608, Taiwan; ^2^ Department of Oral and Maxillofacial Surgery, Mackay Memorial Hospital, Taipei 10449, Taiwan; ^3^ Institute of Oral Biology, School of Dentistry, National Yang-Ming University, Taipei 11221, Taiwan; ^4^ School of Chinese Medicine, China Medical University, Taichung 40402, Taiwan; ^5^ Department of Medical Research, China Medical University Hospital, Taichung 40402, Taiwan; ^6^ Department of Medical Research, Mackay Memorial Hospital, New Taipei City 25160, Taiwan; ^7^ Department of Chemical Engineering and Biotechnology, National Taipei University of Technology, Taipei 10608, Taiwan; ^8^ Institute of Biomedical Engineering and Nanomedicine, National Health Research Institutes, Miaoli County 35053, Taiwan; ^9^ Department of Radiation Oncology, Mackay Memorial Hospital, Taipei 10449, Taiwan

**Keywords:** BI2536, mitotic catastrophe, oral cancer, PLK1, radiosensitization

## Abstract

BI2536 has been developed as a potential therapeutic agent for various cancers but not in oral cancer cells. Since BI2536 exhibits mitosis-regulating activity which are the most radiosensitive, we hypothesized that BI2536 might modulate the radiosensitivity of oral cancer cells. Human normal fibroblasts, oral cancer SAS, and OECM1 cells were treated with BI2536 (0–50 nM) and/or radiation (0–4 Gy). MTT assay, Liu's staining, flow cytometry, clonogenic assay, Annexin V/propidium iodide (PI) staining, western blot analysis, and small interfering RNA knockdown experiments were used to assess cell viability, morphology, cell cycle progression, radiation survival, and expression of regulatory proteins *in vitro*. Male BALB/c nude mice implanted with SAS cells were used to examine the effects of BI2536 *in vivo*. Treatment with BI2536 preferentially inhibited the viability of SAS and OECM1 cells, but not the normal fibroblasts. Morphological examination and Annexin V/PI staining of BI2536-treated oral cancer cells showed mitotic catastrophe and apoptosis. A DNA histogram revealed BI2536 induced G2/M and upregulation of phosphorylated H3 indicating accumulation in the M phase. BI2536 modulated the expression of PLK1, cell division control protein (Cdc)2, Cdc20, Cdc25c, adenomatous polyposis coli 3, and cyclin B1. At 10 nM, BI2536 exhibited low cytotoxicity, effectively induced mitotic catastrophe, and more importantly, sensitized oral cancer cells to radiotherapy. The animal study showed that BI2536 (10 mg/kg) + radiation (2 Gy) resulted in stronger tumor inhibition than that associated with radiation alone. Our findings showed that BI2536 could be an effective radiosensitizer both *in vitro* and *in vivo*.

## INTRODUCTION

Head and neck cancer represents an important global burden, with more than 500,000 new cases diagnosed annually worldwide [1]. The oral cavity is the most predominant site affected by these cancers [2]. Surgery and radiotherapy alone are considered the curative treatment modalities for oral squamous cell carcinoma (OSCC). However, loco-regional recurrent rates are approximately 20 to 50% for patients who undergo radical treatment during the first two years [3]. Radiotherapy is important for management of the unfavorable pathological features after surgery and salvage treatment against persistent or recurrent OSCC. To improve the survival rate of patients with OSCC, cisplatin may be used as a radiosensitizer. The current use of cisplatin only provides moderate control of OSCC. Therefore, it is important to develop a novel radiosensitizer to enhance the efficacy of radiotherapy in treating oral cancer.

Strategies for radiosensitization include S phase cell cycle checkpoint dysregulation in tumor cells, DNA repair inhibition, and cell cycle arrest at the G2/M phase [4]. Because cells at the G2/M phase, particularly M phase, are the most radiosensitive, pharmacological agents with microtubule-interfering activity, such as paclitaxel, are promising radiosensitizers [5].

Polo-like kinase 1 (PLK1) is an important member of the serine/threonine protein kinase family. It has been shown to play various roles in the regulation of mitosis and throughout the cell cycle [6]. It controls mitotic entry, centrosome separation and maturation, chromosome arm resolution, microtubule kinetochore attachment, spindle assembly checkpoint (SAC) silencing, and cytokinesis [7]. Anti-PLK1 antibodies and small interfering RNA (siRNA) treatment-based studies have shown the essential role of PLK1 in the mitotic progression of cancer [8, 9]. However, its precise mechanism and specific functional role in carcinogenesis and tumor progression is not well understood [10]. Approximately 80% of human tumors express high levels of PLK1 transcripts, whereas PLK1 mRNA is mostly absent in the surrounding healthy tissues [11]. Overexpression of PLK1 was shown to be associated with a poor prognosis in several tumors and a low overall survival rate [12]. Forced overexpression of PLK1 resulted in malignant transformation of normal human fibroblasts *in vitro*, suggesting that PLK1 might directly contribute to carcinogenesis [13]. Thus, many PLK1 inhibitors have been developed as potential cancer therapeutic agents. One of these compounds, BI2536, a PLK1 specific inhibitor, has shown antitumor and cytotoxic effects in several cancers [14, 15]. BI2536 potently binds to the kinase domain of PLK1 and inhibits its activity, including regulation of multiple mitotic processes and cell cycle [16]. Many studies have suggested that BI2536 might be a potential anticancer agent; however, its use as a radiosensitizer in head and neck cancer has not been investigated.

In the present study, we examined the effects of BI2536 on cell cycle regulation and investigated its putative radiosensitizing activity in oral cancer cells.

## RESULTS

### BI2536 preferentially inhibited oral cancer cell viability

**Figure 1:**
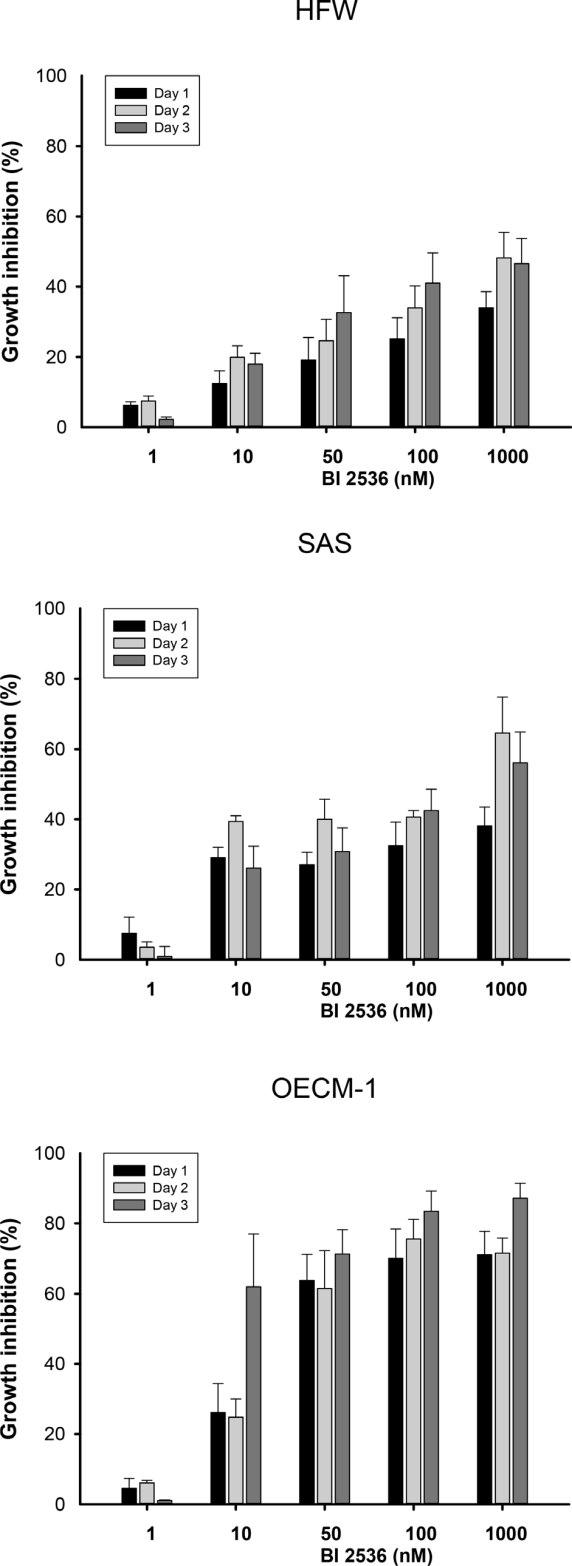
Cell viability of human fibroblasts (HFW) and OSCC (SAS and OECM-1) cells treated with various concentrations of BI2536 Cells were treated with BI2536 at 1–1000 nM for 1–3 days. MTT assay was used to evaluate cell viability. Data are expressed as the means ± SE from three separate experiments.

To investigate the effects of BI2536 in human fibroblasts and OSCC cells, we monitored the viability of HFW, SAS, and OECM-1 cells treated with BI2536. BI2536 inhibited the growth of OSCC cells in time- and concentration-dependent manners. Treatment with BI2536 at concentrations >10 nM for 24 h significantly reduced the growth of SAS and OECM-1 cells, but not that of human fibroblasts (Figure 1). The half maximal inhibitory concentration (IC_50_) values of BI2536 in SAS, OECM-1, and HWF on day 2 were 160, 32, and >1000 nM, respectively. HacaT cells from oral epithelium were used as control cells in this study. There was no significant difference between HacaT and oral cancer cells in the MTT assay. However, the morphological examination showed more mitotic arrest and less mitotic catastrophe in HacaT cells treated with BI2536 (Supplementary Figure 1A and 1B).

### BI2536 treatment induced morphological changes

**Figure 2:**
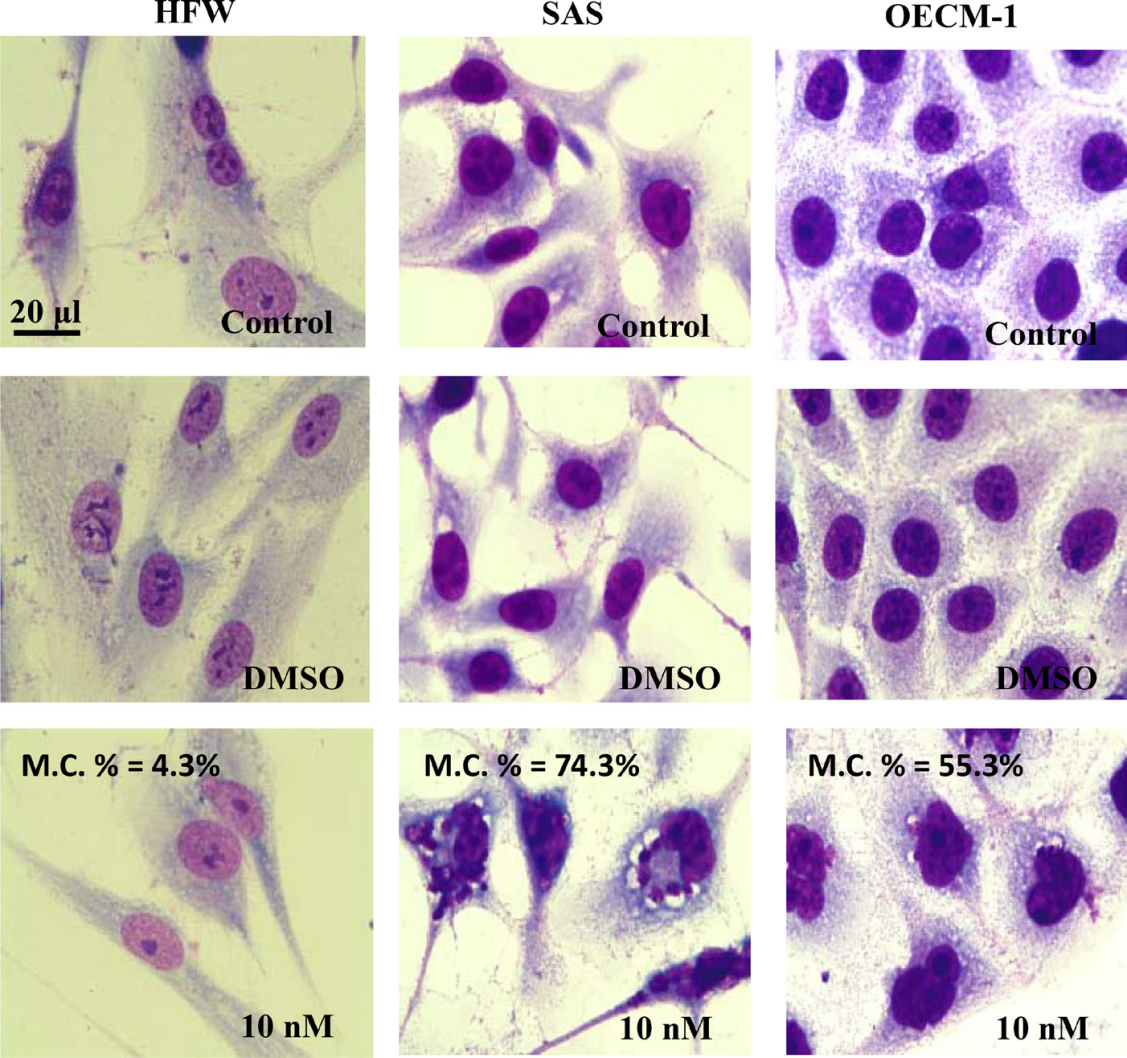
Morphology of HFW, SAS, and OECM-1 cells treated with BI2536 Cells were treated with BI2536 at a concentration of 10 nM, harvested, stained by Liu's stain, and observed under a light microscope. Micrographs were taken at a magnification of 1000×. The ratio of mitotic catastrophe was 4.3, 74.3, and 55.3% in HFW, SAS, and OECM-1 cells. M.C., mitotic catastrophe.

In human fibroblasts, BI2536 treatment at concentrations <10 nM did not affect the cell structure during the first two days. However, the morphology of BI2536-treated oral cancer cells showed typical features of mitotic catastrophe and apoptosis on day 1 even at 1 nM; this became more evident as the concentration and time increased (Figure 2). These findings suggested that BI2536 exhibited antitumor activity and induced tumor cell apoptosis.

### BI2536 arrested oral cancer cells at the M phase

**Figure 3:**
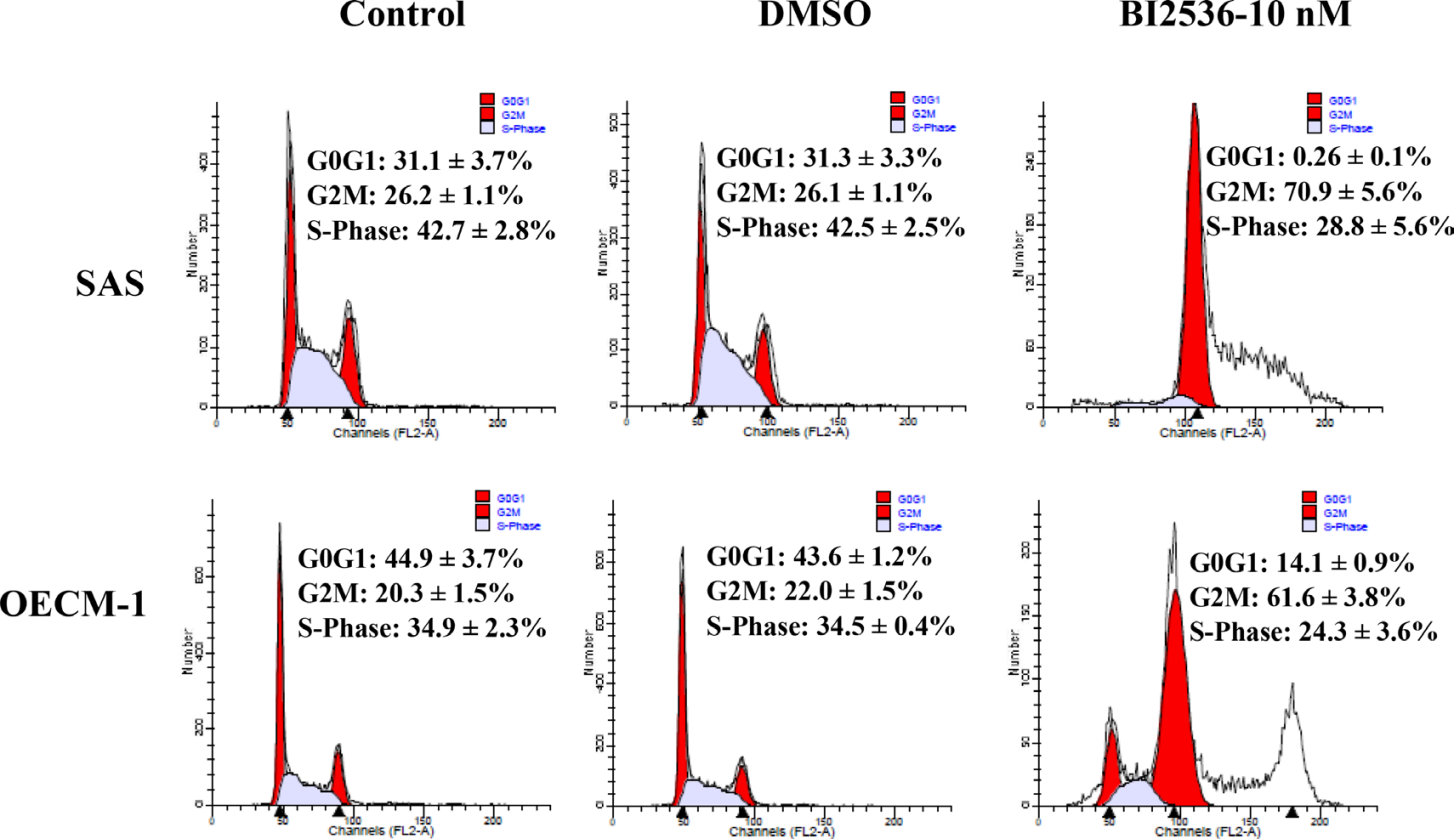
Cell cycle distribution of SAS and OECM-1 cells treated with BI2536 Cells were treated with BI2536 at 10 nM for 24 h. Flow cytometry was performed to analyze the DNA histogram of cell cycle distribution. Treatment of SAS and OECM-1 cells with BI2536 at 10 nM increased the percentage of cells in the G2/M phase.

**Figure 4:**
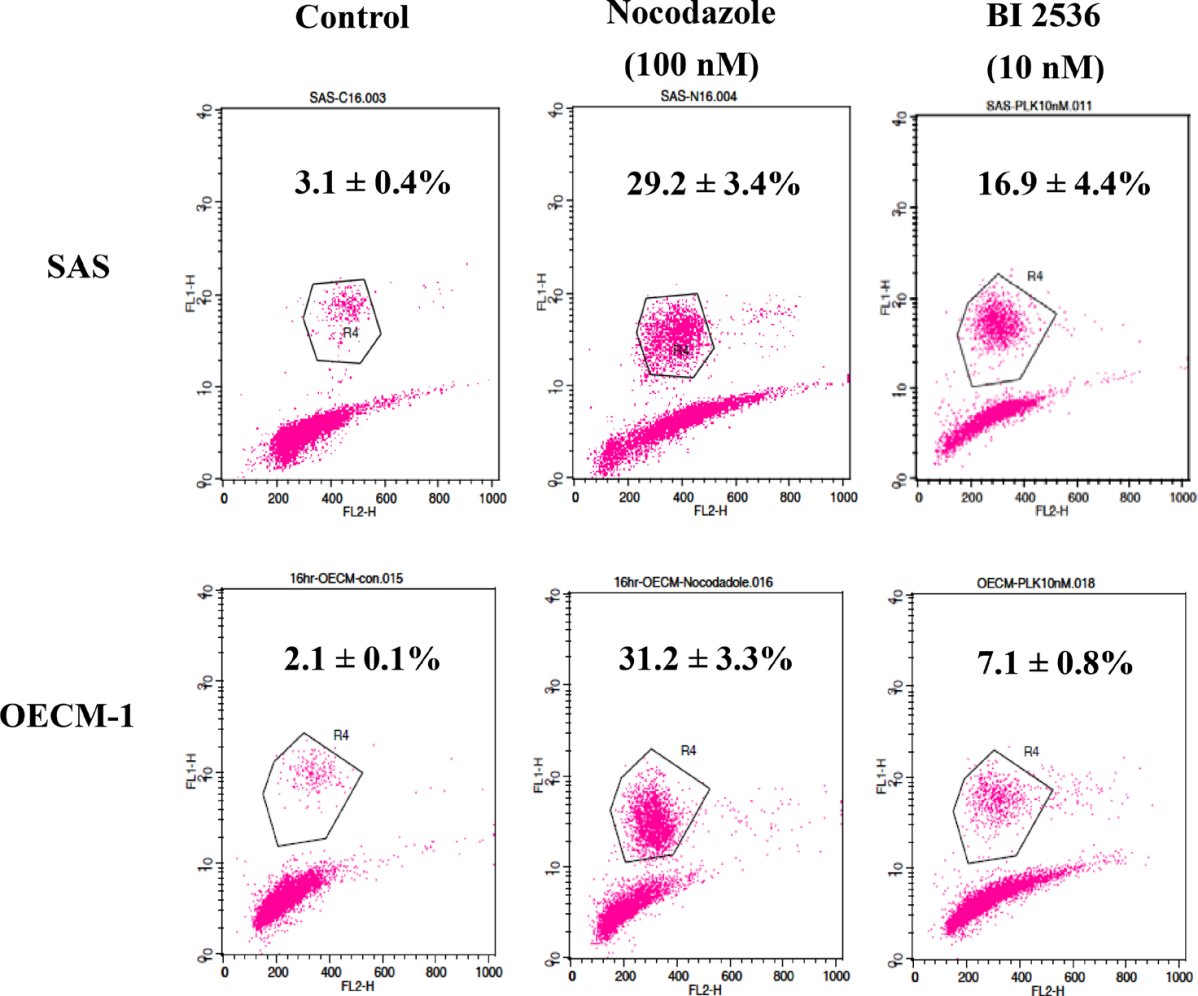
Expression of phosphorylated histone H3 in SAS and OECM-1 cells Cells were treated with nocodazole (100 nM, a positive control) and BI2536 (10 nM) and then collected for flow cytometry. Data are expressed as the means ± SE. Treatment of SAS and OECM-1 cells with BI2536 at 10 nM increased the ratio of cells in the M phase.

The DNA histogram revealed that BI2536 caused G2/M arrest with marked polyploidy and few hypoploid cells (Figure 3). BI2536 treatment resulted in upregulation of phosphorylated H3, indicating cell accumulation in the M phase (Figure 4). In addition, BI2536 induced mitotic arrest of oral cancer SAS and OECM-1 cell lines.

### BI2536 induced apoptosis in oral cancer cells

**Figure 5:**
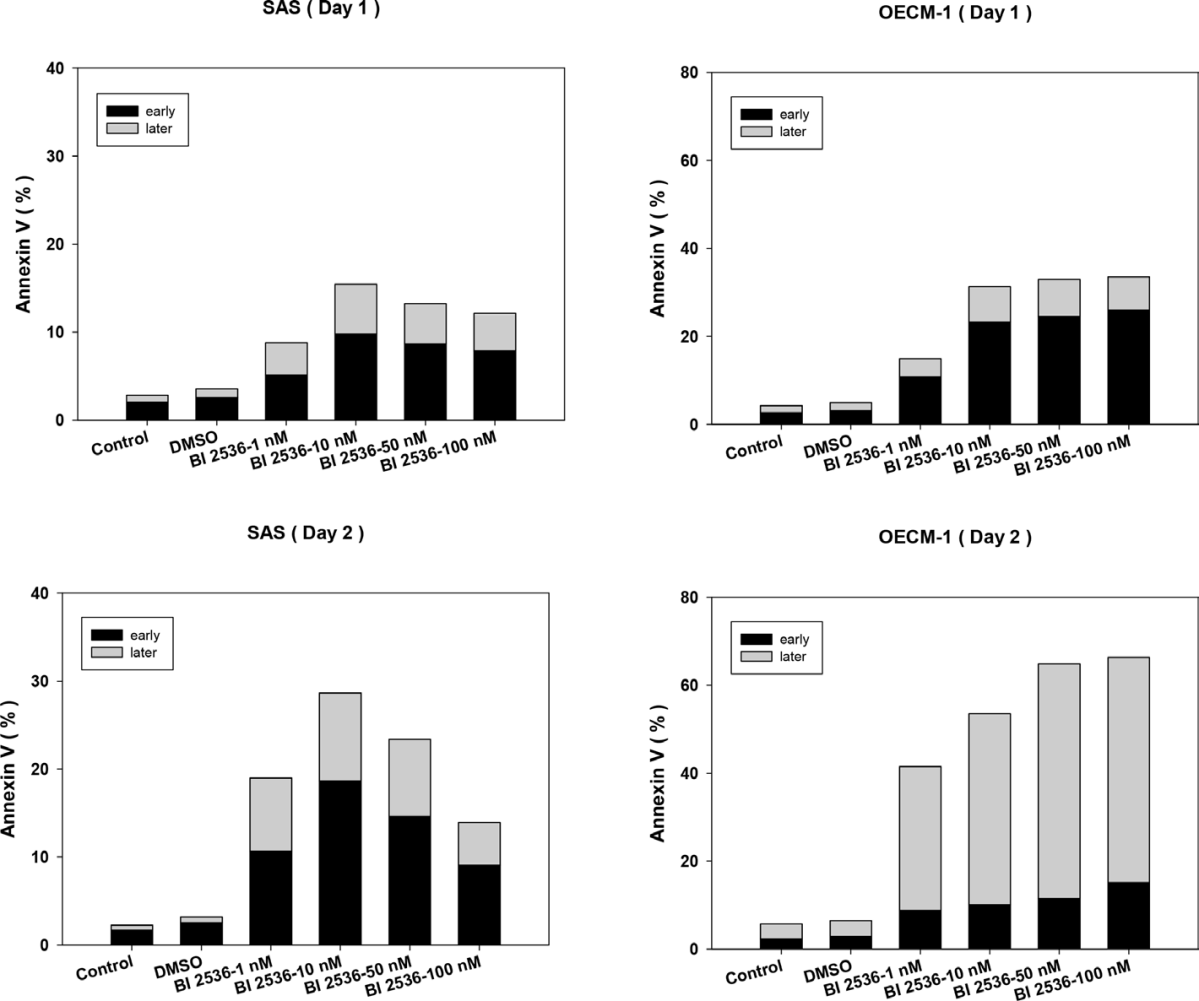
BI2536 induced early and late apoptosis in SAS and OECM-1 cells BI2536 treatment at concentrations >10 nM resulted in a marked increase in both early and late apoptosis.

Annexin V and PI staining was used to detect early and late apoptosis. Cells treated by BI2536 exhibited a marked increase in both early and late apoptosis (Figure 5).

### Expression of proteins related to mitotic arrest

**Figure 6:**
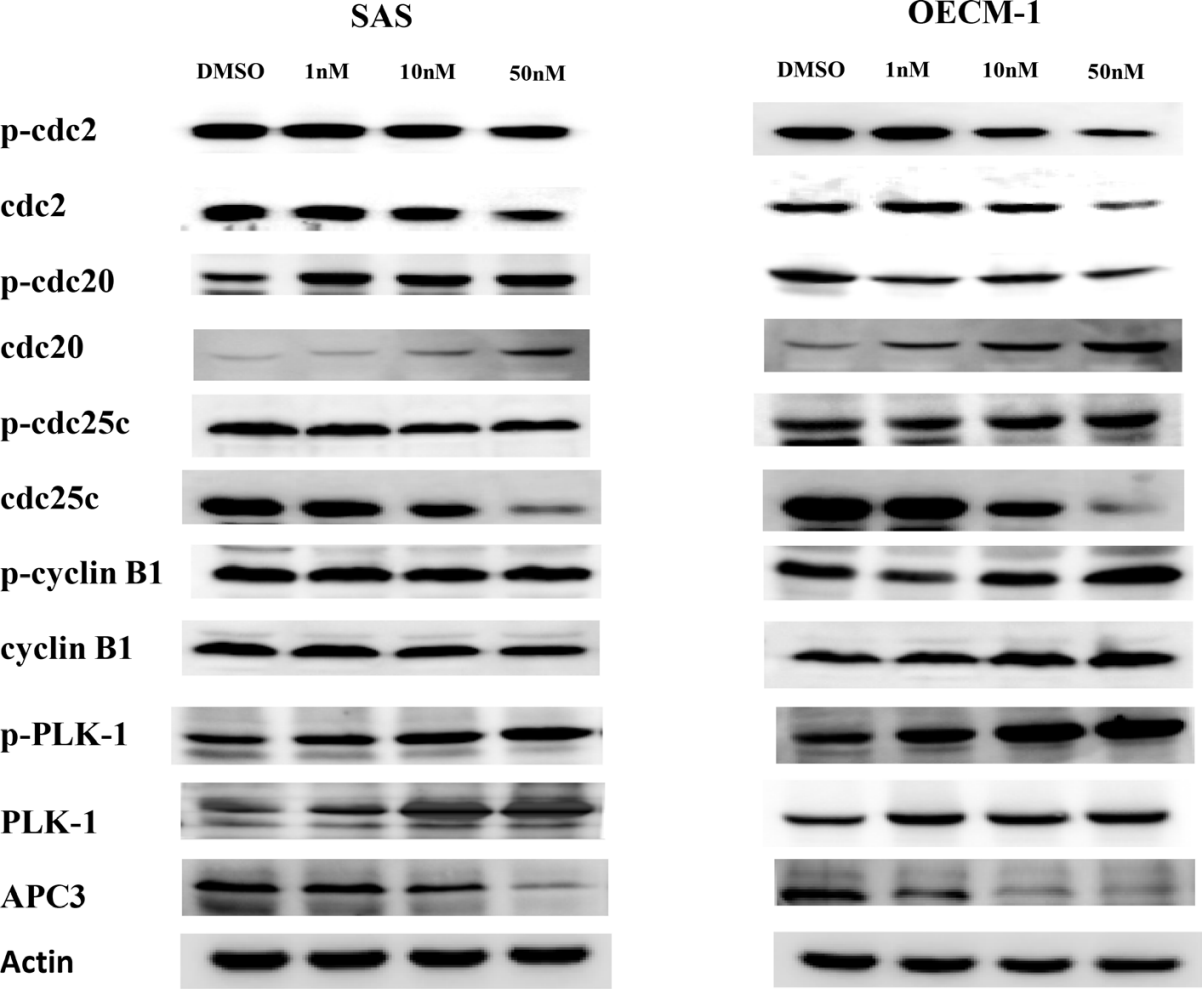
The expression of proteins related to cell cycle regulation in SAS and OECM-1 cells Cells treated with BI2536 at 0 nM, 1, 10, and 50nM were harvested for extraction of proteins. Western blot analysis was performed to measure the protein expression.

**Figure 7:**
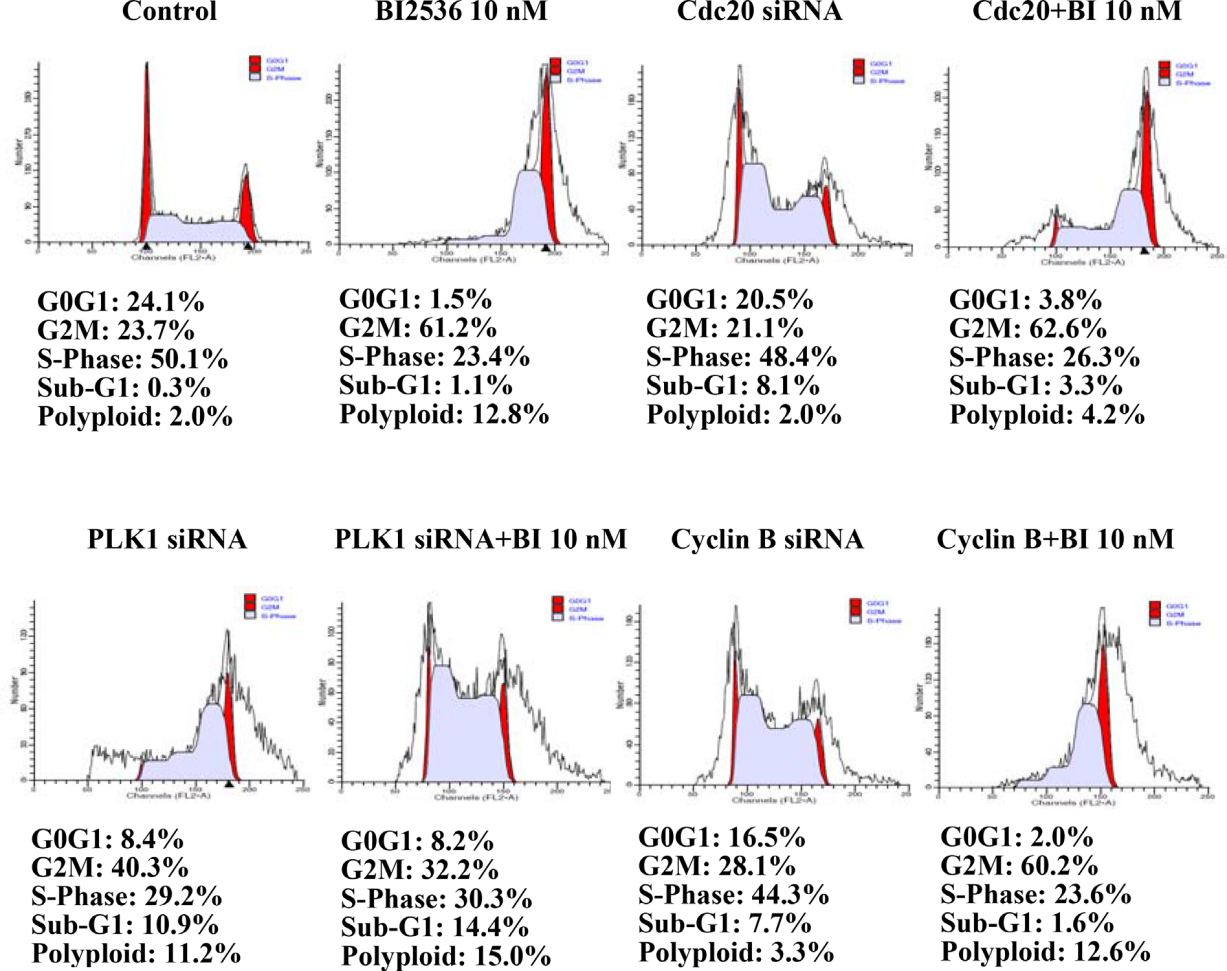
Flow cytometry of SAS cells transfected with siRNA for 24 h PLK1 knockdown could partially reverse the G2/M accumulation induced by BI2536.

Results showed that BI2536 upregulated PLK1, cyclin B1, and cell division control protein (Cdc)20 expression and downregulated Cdc2, Cdc25, and adenomatous polyposis coli 3 (APC3) expression in both SAS and OECM-1 cells. Interestingly, BI2536 upregulated the expression of p-PLK1 in both SAS and OECM1 cell lines at the same concentration. This finding potentially disputes the claimed effects of PLK1 in inhibition of oral cancer (Figure 6). To elucidate BI2536-induced cell cycle alterations, we performed PLK1, Cdc20, and cyclin B knockdown experiments. Results showed that PLK1 knockdown could partially reverse BI2536-induced G2/M accumulation (Figure 7).

### BI2536 sensitized oral cancer cells to radiation

**Figure 8:**
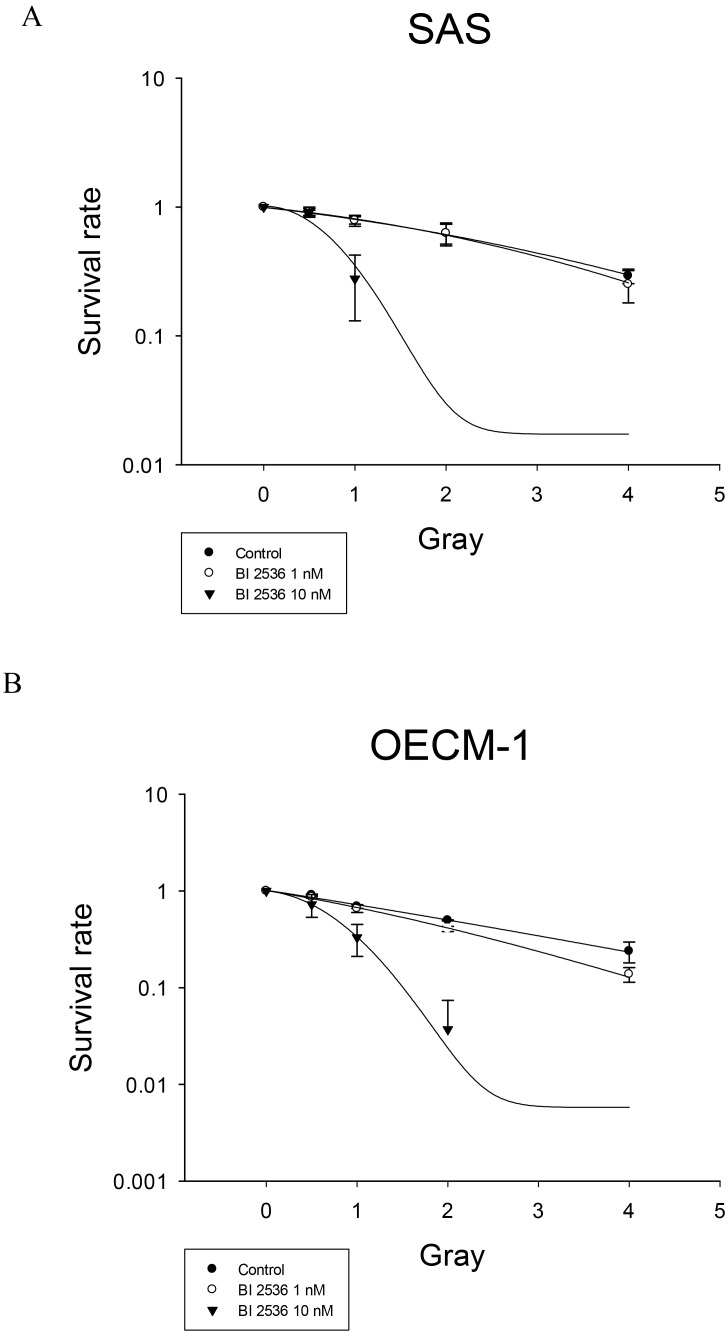
Radiation survival of SAS and OECM-1 cells Cells were treated with 1 and 10 nM BI2536 and subjected to a clonogenic assay. The radiation survival curves were plotted by using the linear quadratic model. (**A**) SAS cells (**B**) OECM-1 cells.

At low cytotoxic concentrations (1 and 10 nM), BI2536 sensitized human oral cancer cells to radiation therapy. In SAS and OECM-1 cells, the sensitizer enhancement ratio (SER) of BI2536 at 1 and 10 nM was 1.06 and 3.55, respectively for SAS cells and 1.27 and 2.93, respectively for OECM1 cells (Figure 8).

### BI2536 inhibited SAS cell growth *in vivo*

**Figure 9:**
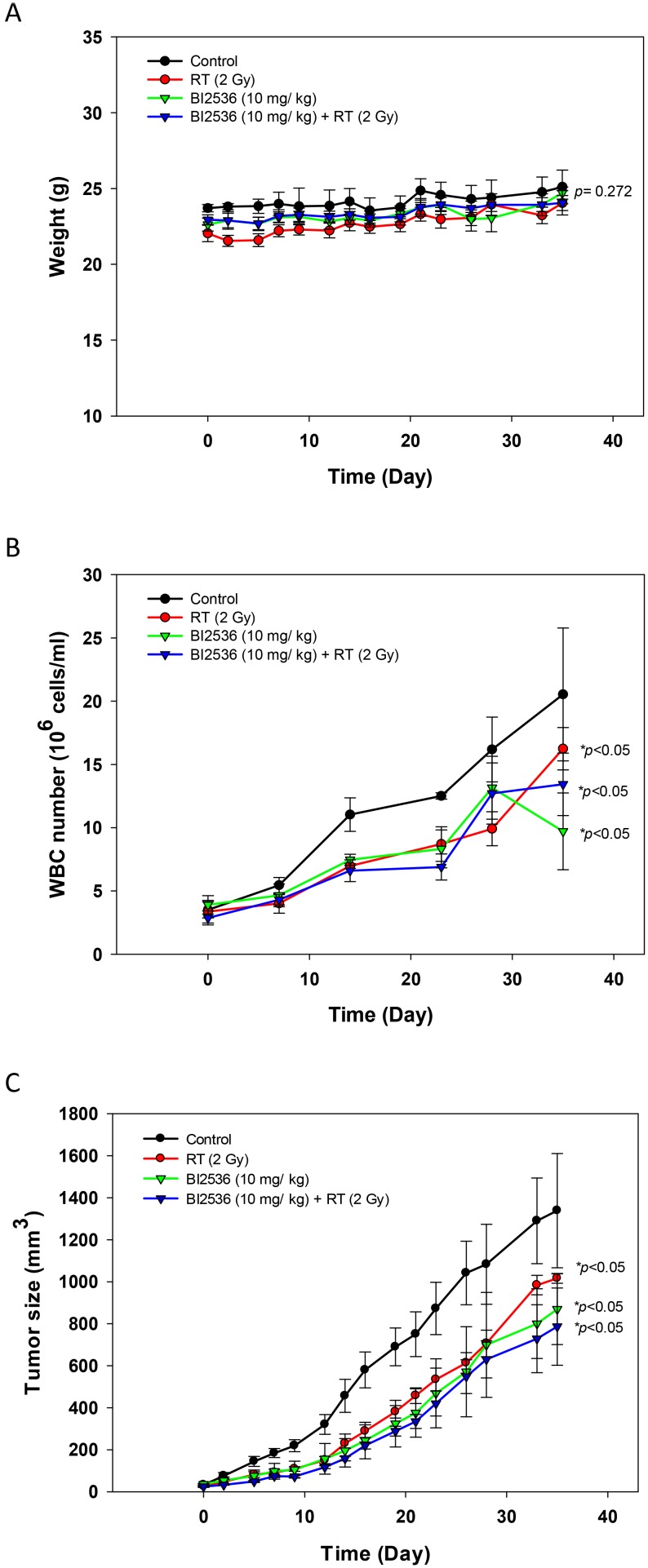
Effects of radiation and BI2536 in SAS xenograft mice (**A**) Body weight; (**B**) WBC; (**C**) tumor volume. ^*^p < 0.05, compared to the control group.

Throughout the treatment period, there was no obvious body weight loss after radiation and/or BI2536 treatment (*p* = 0.272; Figure 9A). The WBC counts of the treated groups (5 × 10^6^ to 20 × 10^6^ cells/mL) were lower than that of the control group (*p* < 0.05) (Figure 9B). Radiation, BI2536 (10 mg/kg), and combination treatment significantly reduced the size of SAS tumor xenograft (*p* < 0.05). Interestingly, BI2536 (10 mg/kg) plus radiation (2 Gy) resulted in stronger tumor inhibition than that of radiation alone (Figure 9C).

## DISCUSSION

The results of this study showed that BI2536 induced G2/M arrest with marked polyploidy and few hypoploid cells. Upregulation of phosphorylated H3 further indicated that BI2536 resulted in accumulation of cells in the M phase, suggesting oral cancer cell cycle arrest in this phase. BI2536-treated cells exhibited a multinucleated morphology, indicating that BI2536 inhibited cell viability by inducing mitotic catastrophe and apoptosis in human oral cancer cells. Annexin V flow cytometry supported that BI2536 induced apoptosis in OSCC cell line. Choi *et al.* showed that BI2536 led to mitotic catastrophe in several non-small cell lung cancer (NSCLC) cell lines *via* prolonged activation of SAC [14]. BI2536 treatment resulted in mitotic arrest because of the improper formation of the mitotic spindles and mitotic centrosomes. The unattached kinetochores in BI2536-treated NSCLC cells resulted in prolonged SAC activation, which in turn led to mitotic catastrophe. Finally, BI2536-treated NSCLC cells exhibited defective proliferation [14]. PLK1 inhibitors, such as BI2536, are potential new anticancer agents. It is important to assess the effects of PLK1 inhibition in combination with conventional treatments, such as radiation. Several studies have shown that PLK1 inhibition combined with radiation leads to synergistic cell killing, suggesting that PLK1 inhibitors together with radiotherapy might be particularly beneficial [17–19].

Cells at the G2 and M phases are more sensitive to radiation than those at other cell cycle phases. BI2536 could inhibit mitosis; therefore, its administration prior to radiation could result in radiosensitization. In this study, BI2536 was found as a potent radiosensitizer at concentrations >10 nM. BI2536 treatment for 24 h before radiation resulted in accumulation of cells in the G2 and M phases, which impaired the repair of radiation-induced damage. However, another study showed that administration of PLK1 inhibitors after radiation might cause radioresistance by prolonging the G2 checkpoint and inducing cell repair [20]. To elucidate the actual effects of BI2536 on radiosensitivity, we examined the radiosensitizing activity of BI2536 in SAS xenograft animal model. BI2536 might act as a potent radiosensitizer of oral cancer cells, accompanied by induction of mitotic catastrophe and mitotic arrest.

Our study showed that the G2/M population significantly increased in BI2536-treated cells, suggesting an increase in the number of mitotic cells. In addition, BI2536 affected cell cycle regulators, which coordinate critical protein kinases and phosphatases in the G2/M phases. PLK1 plays a pivotal role during the M phase and cytokinesis in cancer cells. Cyclin-dependent kinase 1 (CDK1), cyclin B, and APC3 regulate the transition between cell cycle phases, although they are not always the drivers of the process. Instead, they respond to signals within the cell and from the external environment. Our study showed that BI2536 induced mitotic arrest with alteration in the expression of Cdc2, Cdc20, Cdc25, cyclin B1, PLK1, and APC3. In particular, BI2536 treatment induced upregulation of Cdc20, cyclin B1, and PLK1 expression and downregulation of Cdc2, Cdc25, and APC3 expression. However, BI2536 inhibited PLK1 activity, as evidenced by the decrease in the binding and phosphorylation of cdc25. This might explain the coordination between the regulatory activities of CDK1 and cyclin B [21].

Choi *et al.* also showed that BI2536 treatment induced PLK1 overexpression, which activated SAC in NSCLC cells [14]. Despite BI2536-induced PLK1 overexpression, its activity was inhibited. The results of this previous study showed that BI2536 inhibited PLK1 activity and blocked the hyperphosphorylation of the checkpoint protein, BubR1, which is sensitive to kinetochore tension loss. To clarify the mechanism underlying the overexpression of cell cycle proteins in our study, Cdc20, cyclin B1, and PLK1 knockdown was performed in SAS cells using siRNA. Our results revealed that PLK1 knockdown could partially reverse BI2536-induced G2/M accumulation. Thus, PLK1 might mediate BI2536-induced cell cycle alterations. It was suggested that BI2536 inhibited the kinase activity of PLK1 without inhibiting its expression.

To date, the key role of PLK1 in carcinogenesis remains unclear. The dose-limiting toxicities of BI2536 account for its unsatisfactory therapeutic effects in clinical trials [22]; however, it might be used as a potential radiosensitizer of oral cancer cells.

In conclusion, our data showed that BI2536 induced mitotic arrest at the M phase in oral cancer cell lines. In addition, it resulted in significantly enhanced efficacy when combined with radiotherapy. The animal xenograft experiment suggested that BI2536 could be a potent radiosensitizer *in vivo*. Further clinical trials are required to validate the antitumor effects of BI2536 and determine its potential use as a PLK1 inhibitor for oral cancer treatment in the future.

## MATERIALS AND METHODS

### Cell culture

Two different squamous cell carcinoma cell lines, mainly of head and neck origin, and one human fibroblast cell line (HFW) were used in this study. SAS cells were obtained from the American Type Culture Collection (ATCC; Manassas, VA, USA). OECM-1 and HFW cell lines were provided by Professor CJ Liu (Taipei, Taiwan) and TC Lee (Academia Sinica, Taipei, Taiwan). SAS and HFW fibroblast cells were cultured in Dulbecco's modified Eagle's medium (DMEM; Corning, NY, USA), whereas OECM1 cells were cultured in Roswell Park Memorial Institute (RPMI) 1640 medium (Corning, NY, USA) supplemented with 10% fetal bovine serum. Cells were maintained in the exponential growth state at 37° C and supplied with 5% CO_2_.

### MTT assay

SAS, HFW, and OECM1 cells (2.5 × 10^4^ cells/well) were seeded in a 24-well plate containing 0.5 mL of the medium. Cells were treated with various concentrations of BI2536 (0, 1, 10, 50, 100, and 1000 nM). After treatment for 24, 48, and 72 h, 3-(4,5-dimethylthiazol-2-yl)-2,5-diphenyltetrazolium bromide (MTT; 5 mg/mL, 50 μL) was added to each well (total volume = 500 μL) and incubated for 4 h at 37° C. Then, the medium was removed, and 500 μL of dimethyl sulfoxide (DMSO) was added to each well for 30 min to dissolve the formazan crystals. The absorbance of each well was measured using test and reference wavelengths of 570 and 630 nm, respectively. Growth inhibition rate was calculated according to the following equation:

Growth inhibition rate = [(number of viable cells in untreated control – number of viable cells in treated group)/number of viable cells in untreated control] × 100

### Liu's staining

Cells (2 × 10^5^ cells/well) were seeded in 6-well plates and treated with various concentrations of BI2536 (0, 1, 10, 50, 100, and 1000 nM) for 24, 48, and 72 h. Liu's staining was performed by adding solution A for 45 s followed by solution B for 90 s (Liu's stain solution, Muto pure chemicals Co., Ltd., Tokyo, Japan).

### Cell cycle analysis

Cells (2 × 10^5^ cells/well) were seeded in 6-well plates and treated with BI2536 (0, 1, 10, and 50 nM) for 24 h. Then, the cells were fixed with cold 70% ethanol. They were sequentially incubated with 200 μL of RNase (3 mg/mL, Sigma) at 37° C for 30 min and 200 μL of propidium iodide (PI, 1 mg/mL, Sigma) at 4° C for 10 min in dark. The DNA histograms were analyzed using BD FACSCalibur^™^ flow cytometer (BD Bioscience, San Jose, CA, USA). The percentages of cancer cells in the G0/G1, S, and G2/M phases were determined using the Cell Quest software (BD Bioscience, San Jose, CA, USA).

### Phosphorylated histone H3 analysis

Cells (2 × 10^5^ cells/well) were seeded in 6-well plates and treated with BI2536 (0, 1, 10, 50, and 100 nM) for 24 h. Cells were fixed with freshly prepared 4% paraformaldehyde (PFA, pH 7.2) for 15 min and then permeabilized with 1 mL of 1% Triton X-100 for 15 min. They were then incubated with 2 μL of phospho-histone H3 (Ser10) (Cell Signaling, Danvers, MA, USA) for 30 min in dark and 500 μL of PI (1 mg/mL) at 4° C for 10 min in dark. The samples were analyzed using the BD FACSCalibur^™^ flow cytometer.

### Annexin V and PI staining

Cells (2 × 10^5^ cells/well) were seeded in 6-well plates and treated with BI2536 (0, 1, 10, 50, and 100 nM) for 24 h and 48 h. They were resuspended in a binding buffer at 2 × 10^6^ cells/mL. Then, cells were incubated with Annexin V (eBioscience, San Diego, USA) and 5 μL of PI (5 mg/mL) for 15 min in dark. The samples were analyzed with the BD FACSCalibur^™^ flow cytometer.

### Western blot analysis

Whole cell lysates were obtained after treatment of the cells with BI2536 (0, 1, 10, and 50 nM) for 24 h. The membrane was blocked with 5% skim milk and immunoblotted with the primary antibodies at 4° C overnight. The following antibodies were used: PLK1 (Millipore, 05844), p-PLK1 (Abcam, EPR2612), Cdc2 (Cell signaling, 9112), p-Cdc2 (Cell signaling, 9111S), Cdc20 (MBL, K0140-3), p-Cdc20 (Cell signaling, 80385), Cdc25 (MBL, K0200-1), p-Cdc25 (Cell signaling, 9528S), cyclin B (Cell signaling, 4135), p-cyclin B (Cell signaling, 4131S), and APC3 (MBL, K0141-3). Then, horseradish peroxidase-labeled secondary antibodies were added (Chemicon, Single Oak Drive, Temecula, CA, USA), and visualization was performed using enhanced chemiluminescence (T-Pro Biotechnology, New Taipei City, Taiwan).

### Colony formation assays for measurement of radiation survival and sensitizer enhancement ratio (SER)

Single-cell suspensions were prepared in 6-well plates (cell density: SAS, 100 cells/well; OECM-1, 200 cells/well; fibroblasts, 200 cells/well). Cells were treated with BI2536 (0, 1, and 10 nM) for 24 h and then irradiated at doses of 0, 0.5, 1, 2, and 4 Gy. After 7–10 days, cells were fixed and stained with a staining solution (3% crystal violet in 20 % methanol). The number of colonies (>50 cells) was counted. The SER was calculated as the radiation dose needed to achieve a survival fraction of 37% using radiation alone divided by that needed using BI2536 plus radiation (D_0_ in radiobiology) [23].

### siRNA treatment

PLK (HSS108120, Invitrogen), Cdc20 (HSS101650, Invitrogen), and cyclin B1 siRNAs (sc-29284, Santa Cruz) were used for transfection using lipofectamine RNAiMAX reagent (Invitrogen, CA, USA), according to the manufacturers’ instructions). SAS and OECM1 cells were seeded in 6-well plates at a density of 2 × 10^5^ cells/well. Then, they were transfected with siRNAs for 24 h. The transfected cells were treated with the indicated doses of BI2536 for 24 or 48 h.

### Animal model

Six-week-old male BALB/c nude mice were obtained from the National Laboratory Animal Center (Taipei, Taiwan) and maintained in accordance to the institutional ethical committee guidelines. Mice were implanted with 2 × 10^6^ SAS cells into the right gluteal region. When tumor size reached 5 mm^3^, mice were divided into four groups of similar body weight and tumor size, as follows: control, radiation-exposed (2 Gy), BI2536-treated (10 mg/kg), and combination therapy (RT [2 Gy] plus BI2536 [10 mg/kg])-treated groups (*n* = 5, each). BI2536 was dissolved in 0.01 N HCl and administered *i.p.* at 10 mg/kg for three consecutive days per week for five cycles. Weight was recorded thrice/week, and blood samples were collected weekly. We measured the white blood cell (WBC) counts, serum alanine aminotransferase (ALT), and creatinine levels. Mice were anesthetized with ketamine (100 mg/kg, *i.p.*) and xylazine (10 mg/kg) before radiation. The gluteal region was irradiated at a radiation dose of 2 Gy using a linear accelerator. One tissue-equivalent polystyrene plate (1.3-cm thick upward and 5-cm thick downward) was used to provide an adequate build-up. Dosimetry was determined using a N30001 ionization chamber (PTW-FREIBURG, Germany) prior to radiation. Tumor volume and survival were analyzed.

### Evaluation of tumor volume and tumor growth inhibition

The size of implanted tumors and body weight of each mouse were determined every other day by one observer. The largest (a) and smallest (b) diameters were measured using calipers, and tumor volume was estimated as follows: tumor volume = 0.5 × ab^2^.

### Body weight, hematological profile, liver and renal functions

In the *in vivo* experiment, body weight was calculated every other day by one observer. Animals were sacrificed five weeks after radiation. The plasma levels of ALT, creatinine, and WBC were measured using a SYNCHRON LX20 spectrophotometer (Beckman Coulter, San Diego, CA, USA).

### Statistical analysis

Statistical analyses were performed using IBM SPSS Statistics 22 (IBM Co., USA). Data were expressed as the means ± standard error of the mean (SE) or as percentages. Results of the animal experiment were analyzed using mixed-model analysis to compare the repeated measurements of tumor size, body weight, and WBC counts among different groups. *P*-values < 0.05 were considered statistically significant.

## SUPPLEMENTARY MATERIALS FIGURE



## Abbreviations

PLK1: polo-like kinase 1
MTT: 3-(4,5-dimethylthiazol-2-yl)-2
PI: propidium iodide
Cdc: cell division cycle protein
APC: adenomatous polyposis coli
IC_50_: half maximal inhibitory concentration
OSCC: oral squamous cell carcinoma
SAC: spindle assembly checkpoint
siRNA: small interfering RNA
mRNA: messenger RNA
DMEM: Dulbecco's modified Eagle's medium
RPMI: Roswell Park Memorial Institute
DMSO: dimethyl sulfoxide
PFA: paraformaldehyde
SER: sensitizer enhancement ratio
HFW: human fibroblast
NSCLC: non-small cell lung cancer
BubR1: budding uninhibited by benzimidazoles 1-related protein 1
WBC: white blood cell
*i.p.*: intraperitoneal
ALT: alanine aminotransferase
Gy: Gray.
