# Supplementary material for: BI2536 induces mitotic catastrophe and radiosensitization in human oral cancer cells
Source: Oncotarget. 2018 Apr 20;9(30):21231–43. doi: 10.18632/oncotarget.25035 (PMC5940398; doi:10.18632/oncotarget.25035)
Supplement: Supplementary file 1 [file oncotarget-09-21231-s001.pdf]

BI2536 induces mitotic catastrophe and radiosensitization in human oral cancer cells

SUPPLEMENTARY MATERIALS

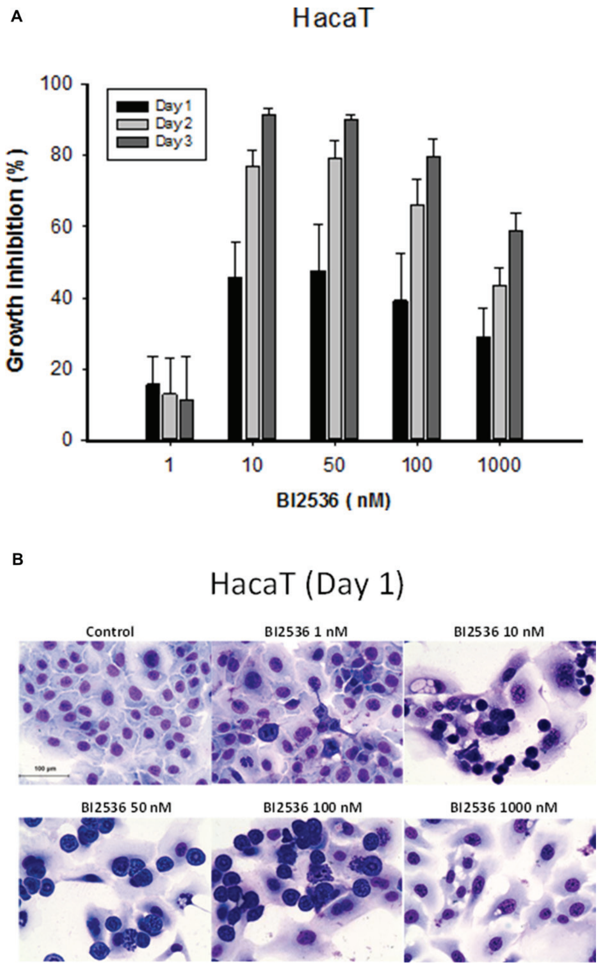

Supplementary Figure 1: Cell viability and morphology of HacaT cells treated with BI2536. (**A**) MTT assay showing growth inhibition of HacaT cells treated with BI2536 (1 to 1000 nM) for 3 days. (**B**) The morphology of HacaT cell treated with BI2536 (1 to 1000 nM) revealed more mitotic arrest and less mitotic catastrophe.
